# Periconceptual Maternal Nutrition Affects Fetal Liver Programming of Energy- and Lipid-Related Genes

**DOI:** 10.3390/ani13040600

**Published:** 2023-02-09

**Authors:** Wellison J. S. Diniz, Alison K. Ward, Kacie L. McCarthy, Cierrah J. Kassetas, Friederike Baumgaertner, Lawrence P. Reynolds, Pawel P. Borowicz, Kevin K. Sedivec, James D. Kirsch, Sheri T. Dorsam, Tammi L. Neville, J. Chris Forcherio, Ronald Scott, Joel S. Caton, Carl R. Dahlen

**Affiliations:** 1 Department of Animal Sciences, Auburn University, Auburn, AL 36849, USA; 2 Department of Animal Sciences, North Dakota State University, Fargo, ND 58108, USA; 3 Department of Animal Science, University of Nebraska-Lincoln, Lincoln, NE 68583, USA; 4 Central Grasslands Research and Extension Center, North Dakota State University, Streeter, ND 58483, USA; 5 Purina Animal Nutrition LLC, Gray Summit, MO 63039, USA

**Keywords:** fetal development, gene expression, hepatic programming, prenatal diet, trace minerals

## Abstract

**Simple Summary:**

Maternal nutrient supply during the periconceptual period has long-term effects on fetal development and tissue function. During pregnancy, the fetus relies on the dam for its nutrient supply. Our objective was to ascertain whether the periconceptual and early life plan of nutrition could affect fetal liver development and gene expression profiles. To this end, we investigated the impacts of maternal vitamin and mineral supplementation (from pre-breeding to day 83) and two rates of body-weight gain during the first 83 days of pregnancy in female fetuses of crossbred Angus beef heifers. We identified 591 unique differentially expressed genes across all six vitamin-gain contrasts. Over-represented pathways were related to energy metabolism, lipid metabolism, and mineral and amino acid transport. Our findings suggest that periconceptual maternal nutrition affects fetal hepatic function through altered expression of energy- and lipid-related genes.

**Abstract:**

During pregnancy, the fetus relies on the dam for its nutrient supply. Nutritional stimuli during fetal organ development can program hepatic metabolism and function. Herein, we investigated the role of vitamin and mineral supplementation (VTM or NoVTM—at least 71 days pre-breeding to day 83 of gestation) and rate of weight gain (low (LG) or moderate (MG)—from breeding to day 83) on the fetal liver transcriptome and the underlying biological pathways. Crossbred Angus beef heifers (*n* = 35) were randomly assigned to one of four treatments in a 2 × 2 factorial design (VTM_LG, VTM_MG, NoVTM_LG, and NoVTM_MG). Gene expression was measured with RNA-Seq in fetal livers collected on day 83 ± 0.27 of gestation. Our results show that vitamin and mineral supplementation and rate of weight gain led to the differential expression of hepatic genes in all treatments. We identified 591 unique differentially expressed genes across all six VTM-gain contrasts (FDR ≤ 0.1). Over-represented pathways were related to energy metabolism, including PPAR and PI3K-Akt signaling pathways, as well as lipid metabolism, mineral transport, and amino acid transport. Our findings suggest that periconceptual maternal nutrition affects fetal hepatic function through altered expression of energy- and lipid-related genes.

## 1. Introduction

From humans to livestock, maternal nutrition has long been recognized as essential for pregnancy success and fetal development [1,2]. During pregnancy, a complex and coordinated array of physiological and metabolic changes occur to support the dam’s metabolism and the growing fetus [3,4]. Likewise, there are critical periods of development in which environmental stimuli lead to changes in embryonic genome expression and programming [2,3,5]. Once programmed, changes in early life may have long-lasting effects in adulthood and may even be transmitted to subsequent generations [6,7]. During this period, nutrient imbalances induce both responsive and adaptive changes in fetal development [8], which may have detrimental or beneficial effects on organ structures and functions, leading to altered metabolism later in life [5,9,10].

Several major events occur during early gestation, including organogenesis [4,10]. Compelling evidence has shown that organs are programmed in utero for postnatal growth and adult function [5,10]. The liver is formed during early gestation and plays a central role in maintaining metabolic homeostasis [11,12,13]. The developing fetal liver is modulated by several biological mechanisms, including gene expression [11]. Through nutritional cues, nutrient-sensing pathways lead to the activation or repression of genes that, in turn, regulate cellular growth and differentiation [8,11]. Although fetal nutritional requirements during early gestation are negligible compared to maternal needs [14], there are likely transitory increases in specific nutrients essential for optimal embryonic and early fetal development [10]. Thus, specific nutrient-supplementation programs during early gestation provide opportunities to offset deficiencies and improve nutrient supply to enhance the fitness of offspring [10,15].

A growing number of studies using ewes and cows have highlighted the effects of maternal nutrition during mid- to late gestation in fetal liver development and function [16,17,18,19]. Prezotto et al. reported increased liver weight in fetuses from nutrient-restricted and re-alimented beef cows compared to the control group [20]. Similarly, maternal nutrient restriction in ewes affected fetal liver morphology, gene expression, and lipid metabolism [16]. Recently, more attention has been directed toward the periconceptual period [19,21,22]. Previously, we have shown that global nutrient restriction in beef heifers from breeding to day 50 of gestation negatively affected fetal liver development and function [21,23]. Altered hepatic gene expression and organ development were reported in response to maternal periconceptual protein restriction in beef heifers [19]. These studies reinforce the roles of macronutrients and maternal diet in programming fetal development and function during gestation. Despite the importance of micronutrients, such as vitamins and minerals, in metabolism, there is still a lack of information regarding their effects on fetal development.

Vitamins and minerals are required in small quantities and are essential in key metabolic processes, such as energy metabolism, DNA synthesis, and gene-expression regulation [3,24,25]. Despite their importance in embryonic and fetal development (reviewed in [25,26,27]), cattle producers still have not widely adopted vitamin and mineral supplementation strategies [28]. Likewise, their role in fetal development during the periconceptual period and early gestation have still to be examined. Thus, our research group developed a model to investigate the role of vitamin and mineral supplementation (VTM or NoVTM—at least 60 days pre-breeding to day 83 of gestation) and two different rates of weight gain (low (LG) or moderate (MG)—from breeding to day 83) on maternal and fetal outcomes [29]. We have shown that nutritional management affects maternal and fetal development [30]. Fetuses from VTM-supplemented dams had increased levels of some trace minerals [30], while managing dams for low body-weight gain led to increased fetal liver size [29]. These changes were likely mediated by the differential expression and regulation of nutrient-transport genes in the placenta [31,32]. Furthermore, changes in nutrient availability affected the fetal liver metabolome, regarding which Crouse et al. reported that the energy metabolism superpathway was differentially affected by the main effect of VTM [33].

To further expand on the previous findings, the current study is based on the hypothesis that periconceptual maternal vitamin and mineral supplementation and moderate rates of weight gain would affect fetal metabolism through differential transcript abundance of genes associated with hepatic metabolism and function. Therefore, we aimed to identify differentially expressed genes (DEGs) in fetal livers, biological processes (BPs), and pathways underlying hepatic function and metabolism in response to periconceptual maternal nutrition. Herein, we report that maternal vitamin and mineral supplementation and rate of body-weight gain in heifers during the periconceptual and early gestation periods affected fetal liver gene expression. These genes regulate pathways such as energy and lipid metabolism and nutrient transport.

## 2. Materials and Methods

### 2.1. Ethics Statement

All animal experiments followed the relevant guidelines and regulations. The experimental design, animal management, and tissue collection were approved by the North Dakota State University Institutional Animal Care and Use Committee (IACUC #A19012).

### 2.2. Animal Model, Experimental Design, and Tissue Collection

The development of the experimental model and study design was previously reported by Menezes et al. [29] and McCarthy et al. [30]. In brief, crossbred Angus beef heifers with average initial body weights = 359.5 ± 7.1 kg were randomly assigned to 1 of 4 treatments in a 2 × 2 factorial arrangement. The factors examined included vitamin and mineral supplementation (VTM or NoVTM) and rate of weight gain (low gain (LG—0.28 kg/day) or moderate gain (MG—0.79 kg/day)). The nutritional management and diet composition per treatment were previously reported elsewhere [29,30]. In brief, the diet consisted of triticale hay, corn silage, modified distiller’s grains plus solubles, ground corn, and, if indicated by treatment, VTM premix. Diets were delivered once daily via a total mixed ration (TMR) in an electronic head-gate facility (American Calan; Northwood, NH, USA). Heifers on the LG treatment were maintained on the basal TMR, whereas heifers under MG were supplemented with a blend of ground corn, dried distiller’s grains plus solubles, wheat midds, fish oil, urea, and ethoxyquin top dressed on the TMR at 0.58% of BW as-fed daily. Diet compositions are described in the Supplementary Materials (Table S1). Heifers were weighed weekly, and feed intake was adjusted during the study to achieve the targeted body-weight gains.

The VTM treatment began at a minimum of 71 days before artificial insemination (AI) by supplementing the heifers with 113 g·heifer^−1^·d^−1^ of a pelleted mineral premix (Purina^®^ Wind & Rain Storm All-Season 7.5 Complete). The VTM supplement provided macro- (calcium, magnesium, phosphorus, potassium, and sodium) and trace minerals (cobalt, copper, iodine, manganese, selenium, and zinc) and vitamins A, D, and E to meet 110% [29] of the requirements, considering the heifers’ physiological requirements [34]. Heifers in the NoVTM group received a pelleted carrier product fed at 0.45 kg/heifer/day with no added vitamins or minerals.

Heifers were estrus-synchronized [35] and bred by AI using female-sexed semen from a single sire. Pregnancy diagnosis was performed 35 days after AI, and fetal sex was determined on day 65 using transrectal ultrasonography [31,36]. At AI and within VTM groups, heifers were assigned to one of two rates of gain—LG or MG. Thus, the arrangements of treatments were as follows: (1) no vitamin and mineral supplementation and low gain (NoVTM_LG, *n* = 9); (2) vitamin and mineral supplementation and low gain (VTM_LG, *n* = 9); (3) no vitamin and mineral supplementation and moderate gain (NoVTM_MG, *n* = 9); and (4) vitamin and mineral supplementation and moderate gain (VTM_MG, *n* = 8).

The treatments continued until day 83 ± 0.27 of gestation. We focused this study on the end of the first trimester of gestation because in this period the placenta is still growing exponentially [37]. Furthermore, fetal organogenesis has already finished and secondary myogenesis is beginning [38]. The fetus was removed through ovariohysterectomy [39] and dissected as previously described [21]. The fetal liver was weighed, and an aliquot was snap-frozen on dry ice and stored at −80 °C.

### 2.3. RNA Isolation, Library Construction, Sequencing, and Data Processing

Hepatic total RNA was isolated using the RNeasy Plus Universal Mini Kit (Qiagen^®^, Germantown, MA, USA), according to the manufacturer’s protocol. The Agilent 2100 Bioanalyzer and agarose gel electrophoresis were used for sample integrity and purity analyses. Based on these, 31 samples (*n* = 8 per group, except VTM_MG—*n* = 7) met the quantity and quality parameters for library preparation. Library preparation and sequencing were carried out by Novogene Co., Ltd. (Nanjing, China). Strand-specific RNA libraries were prepared using the NEBNext^®^ Ultra™ II Directional RNA Library Prep Kit for Illumina (New England BioLabs^®^, Ipswich, MA, USA). Libraries were sequenced on the Illumina^®^ NovaSeq 6000 platform at 150 bp reads and a depth of 20 M reads/sample.

Data cleaning was based on filtering out sequencing adaptors and reads with a PhredScore lower than 30. Quality-control and read statistics were estimated with FastQC v0.11.8 [40]. The FastQC reports were aggregated by MultiQC v1.9 [41]. Cleaned reads were mapped to the *Bos taurus* reference genome (ARS-UCD 1.2) [42] using the STAR aligner v. 2.7.3a [43] and the gene annotation file (release 100) from the Ensembl database. MultiQC, NOISeq v.2.26.0 [44], and edgeR v.3.24.3 [45] were used for post-mapping quality control.

### 2.4. Differential Gene Expression, k-Means Clustering, and Functional Over-Representation Analyses

Read counts from STAR were merged and an expression dataset was created using edgeR. Based on the edgeR *filterByExpr* function [46], genes not expressed or lowly expressed were filtered out. Differential gene expression analysis was carried out using edgeR. This approach applies generalized linear models (GLMs) based on the negative binomial distribution while accounting for normalization factors for different library sizes [45]. To make all pairwise comparisons between the four treatment groups, six contrasts were created as follows: (1) VTM_MG vs. NoVTM_LG, (2) VTM_MG vs. VTM_LG, (3) VTM_MG vs. NoVTM_MG, (4) VTM_LG vs. NoVTM_LG, (5) VTM_LG vs. NoVTM_MG, and (6) NoVTM_MG vs. NoVTM_LG. Differentially expressed genes (DEGs) were identified after multiple-testing correction of the *p*-values based on the Benjamini–Hochberg methodology (FDR ≤ 0.1).

To visualize the intersections among different lists of DEGs, we used UpSet plots created using the UpSetR v.1.4.0 package [47]. To identify over-represented biological processes and pathways underlying the DEGs, we used the ShinyGo v.0.76.2 web tool [48]. Significant results were identified after *p*-value multiple-testing correction (FDR ≤ 0.05).

We investigated the expression patterns of genes with similar behavior to identify biological processes involved with tissue development and function. Genes with less than 1 CPM in at least 50% of the samples were filtered out. The data were normalized through the variance-stabilizing transformation (*VST*) and adjusted for the effect of treatment using the *removebatcheffect* function in Limma [49]. Normalized genes were sorted by the standard deviations (SDs). The top 2,000 genes were then selected (SD ≥ 0.2) for *k*-means clustering analysis using the iDEP v.96 online tool [50]. Genes were mean-centered and then hierarchically clustered using Pearson’s correlation as distance metrics. Data visualization and cluster functional analysis were performed in iDEP.

## 3. Results

### 3.1. Fetal Liver Growth and Mineral Concentration Is Affected by Maternal Diet

The effects of the diets on dam and fetal development were previously reported [29,30]. Briefly, the VTM treatment did not affect the performance or dry-matter intake of the dams. However, the rate of gain (LG vs. MG) led to increased dam body weight at day 83 [29]. Although most fetal morphometric traits were not affected by maternal diet, VTM supplementation resulted in greater fetal liver and intestine weights [29]. Likewise, fetuses from VTM-supplemented dams showed greater concentrations of Se, Cu, and Co in the liver (*p* ≤ 0.05) [30]. Conversely, Mo and Co had greater concentrations in the livers of fetuses from LG dams (*p* ≤ 0.05) [30].

### 3.2. Maternal Diet Affects Fetal Liver Gene Expression

**Figure 1:**
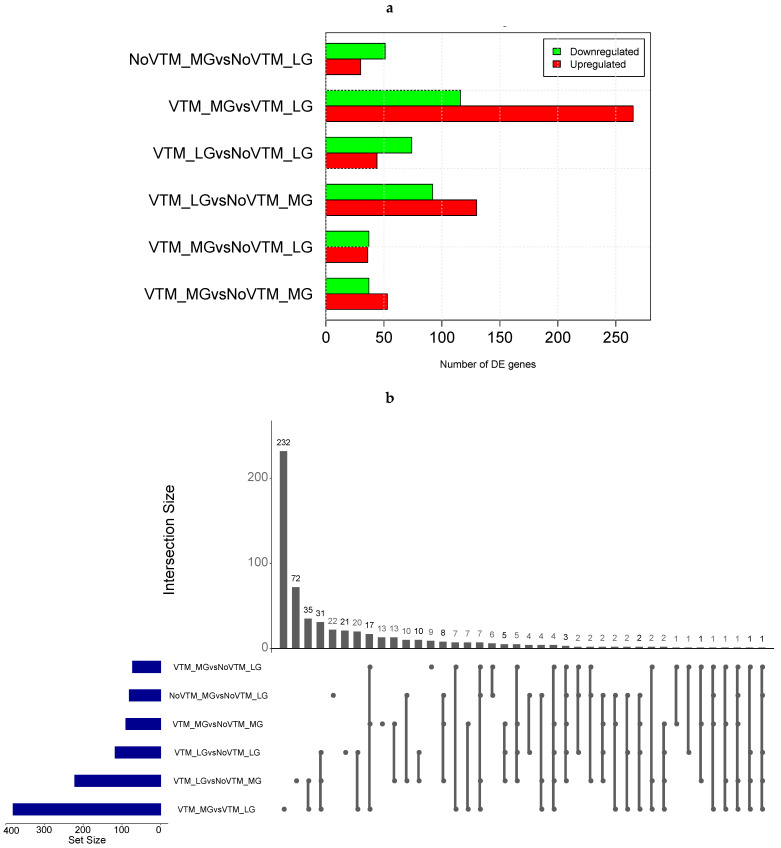
Differential gene expression in the livers of fetuses from heifers receiving or not receiving vitamin–mineral supplementation (VTM or NoVTM) and fed to achieve different rates of gain (low gain (LG) or moderate gain (MG)) during early gestation. (**a**) Number of differentially expressed genes (DEGs) across contrasts. (**b**) The UpSet plot represents the intersection between the sets of DEGs from different contrasts. Each vertical bar shows the number of genes in the intersection. The dot plot reports the set participation in the intersection, and the horizontal bar graph reports the set sizes (totals of DEGs). The treatments were arranged as follows: NoVTM_LG—no vitamin and mineral supplementation and low gain; VTM_LG—vitamin and mineral supplementation and low gain; NoVTM_MG—no vitamin and mineral supplementation and moderate gain; and VTM_MG—vitamin and mineral supplementation and moderate gain.

We profiled the liver transcriptomes of 31 female fetuses at day 83 of gestation from heifers receiving one of four treatments comprising the main effects of vitamin and mineral supplementation (VTM or NoVTM) and rates of weight gain (LG or MG). We generated, on average, 21.45 M of cleaned reads per sample (range from 19.9 to 27.0 M). After QC, sequencing reads were mapped to the ARS-UCD 1.2 reference genome using STAR software. On average, 94.5% of the total reads were uniquely mapped (Supplementary Table S2). After filtering for not-expressed or lowly expressed genes, 14,143 genes were used for differential expression analysis. We identified 591 DEGs across all six contrasts (FDR *≤* 0.1) (Figure 1a). Among them, we identified 4 genes from the ABC transporter family (*ABCA6*, *ABCC2*, *ABCA1*, and *ABCB11*) and 20 from the SLC transporter family, including *SLC27A6, SLC2A2*, and *SLC2A4*, most of them upregulated in the VTM-supplemented groups.

The comparison yielding the greatest number of DEGs was for heifers receiving the vitamin and mineral supplementation but managed at different rates of gain. In this comparison, we found 381 DEGs, with 265 up- and 116 downregulated, in the VTM_MG group compared with the VTM_LG group. We identified selenium-related—*SELENOI* and *SEPSECS*—and zinc-related genes—*MT1E*—upregulated in the VTM_MG group. In the LG group, VTM supplementation resulted in the up- and downregulation of 44 and 74 genes, respectively. The numbers of DEGs overlapping the main effects of VTM or rate of gain (LG, MG) are shown in Figure 1b. The greatest numbers of shared genes were observed between VTM_LG and NoVTM_LG and between VTM_MG and VTM_LG (*n* = 65). Furthermore, we identified bta-mir-2887-1 as being downregulated in four of the contrasts (VTM_LG vs. NoVTM_LG, VTM_LG vs. NoVTM_MG, VTM_MG vs. NoVTM_LG, and VTM_MG vs. NoVTM_MG). Likewise, bta-mir-122 was upregulated in the VTM_LG vs. NoVTM_MG contrast and downregulated in VTM_MG vs. VTM_LG. The DEGs for each comparison with the fold-change values and the overlapping genes are reported in Supplementary Tables S3–S9.

### 3.3. Hepatic Metabolic Pathways, Mineral Absorption, and Amino Acid-Transport Genes Are Affected by Maternal Diet

**Figure 2:**
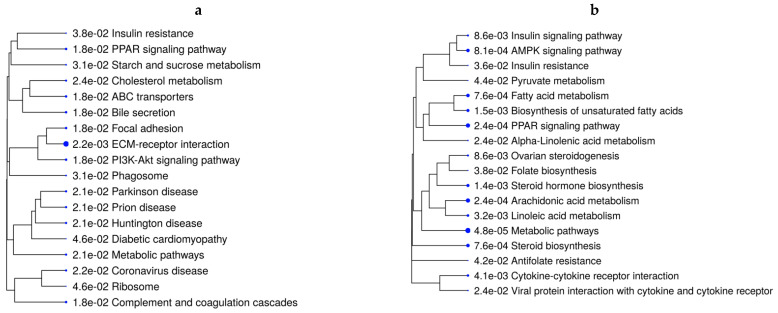
Functional over-representation analysis of differentially expressed genes (DEGs) from fetal liver tissues. KEGG pathways over-represented are based on the DEGs of VTM_MG vs. VTM_LG (**a**) and NoVTM_MG vs. NoVTM_LG (**b**) comparisons. The terms are hierarchically arranged based on functional similarity. The bigger the blue dot, the more significant the term is (FDR ≤ 0.05).

The over-representation analysis of the DEGs to identify pathways retrieved 67 significant KEGG pathways across all contrasts (44 unique pathways) (FDR ≤ 0.05). Supplementary Table S10 reports all unique and overlapping pathways across comparisons based on ShinyGo analysis. Interestingly, the mineral-absorption pathway was over-represented in all VTM-supplemented groups, regardless of the rate of gain, in comparison with no VTM supplementation. Genes in this pathway included *MT1A*, *MT1E*, and *MT2A*, which were upregulated in fetuses receiving VTM supplementation (Table S9). In addition to metabolic pathways over-represented in five of the comparisons, lipid- and energy-metabolism-related pathways, including *PPAR* signaling, PI3K-Akt signaling, AMPK signaling, and insulin resistance, were significant (Table S10). Figure 2a,b show the pathways over-represented by DEGs in response to the rate of gain for each vitamin–mineral supplement condition (VTM_MG vs. VTM_LG and NoVTM_MG vs. NoVTM_LG). Interestingly, lipid-related genes, including *LPL*, *ACSS2*, *FADS2*, *FADS1*, and *FASN*, were downregulated in the MG groups, regardless of the VTM treatment. Furthermore, the genes *INSIG1*, *SLC2A4*, and *SREBF1* were downregulated in the NoVTM_MG vs. NoVTM_LG contrast. On the other hand, *PPARG* and *PPARD* were up- and downregulated, respectively, in VTM_MG vs. VTM_LG. Several unique pathways were over-represented by the DEGs from the NoVTM_MG vs. NoVTM_LG comparison (Figure 2a), including pyruvate metabolism, linoleic acid metabolism, and folate biosynthesis. The KEGG pathways and biological processes (BPs) identified for each of the comparisons, underlying genes, and significance values are provided in Tables S3–S9.

**Figure 3:**
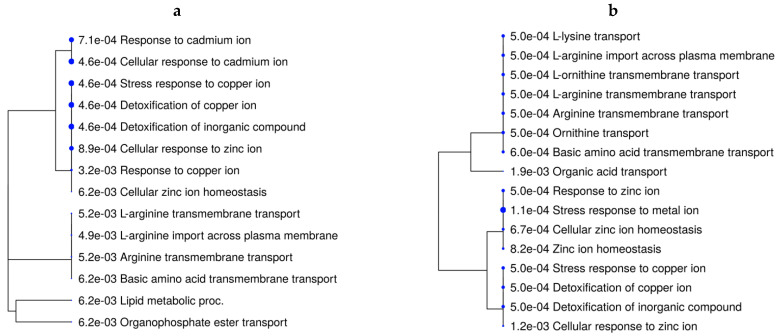
Functional over-representation analysis of differentially expressed genes in fetal liver tissues. Biological processes over-represented are based on the DEGs of VTM_MG vs. NoVTM_MG (**a**) and VTM_LG vs. NoVTM_LG (**b**) comparisons. The terms are hierarchically arranged based on functional similarity. The bigger the blue dot, the more significant the term is (FDR ≤ 0.05).

The over-represented BPs underlying the DEGs from the main effect of rate of gain were related to lipid metabolism, lipid storage, and the fatty acid metabolic process (Tables S3 and S8). When evaluating the effects of vitamin–mineral supplementation for each rate-of-gain condition (VTM_MG vs. NoVTM_MG and VTM_LG vs. NoVTM_LG—Figure 3a,b), mineral-homeostasis- and amino acid-transport-related terms were over-represented in fetuses receiving in utero VTM supplementation.

The *k*-means clustering analysis, followed by functional over-representation, provided insights into the molecular pathways underlying gene expressions and their involvement with liver function. Based on the *k*-means approach, we clustered 2000 genes in four clusters (Table 1). Cluster A grouped 702 genes involved with pathways such as retinol metabolism, arachidonic acid metabolism, and the PPAR signaling pathway (FDR ≤ 0.05). Additional pathways related to metabolism included PI3K-Akt signaling and oxidative phosphorylation, which were identified in clusters B (*n* = 577) and D (*n* = 364), respectively. The genes within each cluster and the *k*-means values are reported in Table S11. The over-represented pathways and genes for all clusters are reported in Table S12 (FDR ≤ 0.05).

## 4. Discussion

As in humans, nutrition in livestock species during pregnancy has long been recognized as affecting maternal metabolism and, consequently, fetal growth and development [51]. Maternal models based on excess or restricted nutrition have shown adverse effects on the availability of nutrients and metabolites to developing fetuses [33,52,53]. During pregnancy, there are physiological changes related to maternal and fetal metabolic requirements [3,10,14]. Cumulative evidence has shown that although fetal growth during early pregnancy is minimal, there is likely increased demand for specific nutrients [10,14,15]. Thus, managing nutrient supply before and during pregnancy is critical for proper fetal development and growth [10,15]. Most studies investigating the effects of maternal nutrition on fetal outcomes have focused on macronutrients, such as proteins and energy, during the last two-thirds of gestation. Lately, we and others have extended our attention to the role of micronutrients during the periconceptual period and early pregnancy.

We have developed a beef cattle model and nutrition plan that simulates on-farm scenarios [29]. Based on this model, we have provided strong evidence that maternal supplementation programs during the periconceptual period affect both maternal and fetal development [29,30]. Changes in nutrient availability lead to maternal adaptive changes, including placental adaptations [15,54,55,56,57]. For the same heifers as those used in the current study, we have reported changes in gene expression in placental caruncle (maternal placental) and cotyledon (fetal placental) tissues [31]. Among the findings, we identified 267 unique DEGs underlying biological processes related to nutrient transport, ion homeostasis, and lipid metabolism [31]. As the placenta is a unit of nutrient transport, it is interesting to note that several of the pathways over-represented in the placenta, including PPAR signaling, insulin resistance, cholesterol metabolism, and ion and amino acid transport, were also identified in fetal livers in the current experiment. Additionally, we reported that maternal rate of gain affected the fetal intestine, liver and femur mass, and lipid and metabolite abundance. Likewise, liver mass and mineral concentration in the fetuses and amino acid abundance in the allantoic fluid [29,30] were affected by mineral and vitamin supplementation. The previous findings reported for the experimental model described here are supported by the differentially expressed genes that we identified. The same trend was also observed for pathways and biological processes.

### 4.1. Maternal Body-Weight Gain during Early Pregnancy Affects the Expression of Hepatic Fetal Genes Involved with Lipid and Energy Metabolism

The fetal liver develops early in gestation and is sensitive to hormonal and nutritional imbalances [11]. Tissue growth and function are programmed in utero by the coordinated function of genes [18]. By clustering the 2000 genes with the greatest expression variation, regardless of maternal diet, we identified several over-represented pathways involved with liver metabolic function. These pathways were mainly related to energy metabolism, including PPAR and PI3K-Akt signaling pathways. Pathways related to tissue development, such as ECM-receptor interaction and focal adhesion, were over-represented. Interestingly, genes acting on these pathways were differentially expressed among the treatments (MG vs. LG).

It is important to note that the maternal rate of weight gain affected both gene expression and tissue development. We have shown that fetuses from the MG group had increased fetal femur weight, suggesting a potential effect on bone growth in the MG vs. LG dams. Findings from Yin et al. [58] indicate that in utero diet influences peak bone mass in adolescents. Likewise, fetuses from heifers with LG showed a heavier total liver mass when compared to the MG group [29]. Fetal hepatic metabolites were also affected by rate of gain, including those involved with carbohydrate and energy metabolism [33]. Several other studies reported the effects of maternal nutrient restriction on fetal hepatic development [11], gene expression [18,21,23], and metabolite abundance [33]. We found bta-miR-122 to be downregulated in the VTM_MG vs. VTM_LG group but upregulated in the VTM_LG vs. NoVTM_MG group. This miRNA acts to regulate cholesterol metabolism and terminal differentiation of hepatocytes [59].

We identified 591 unique DEGs across all six contrasts. However, the effect of rate of gain (LG or MG) combined with VTM supplementation seems to be stronger, as more genes were differentially expressed in this comparison (i.e., VTM_MG vs. VTM_LG). Several genes involved with energy homeostasis and lipid metabolism pathways were downregulated in the VTM_MG compared to the VTM_LG group. Although with a less pronounced effect on the number of DEGs, similar energy-related pathways were over-represented when there was no vitamin and mineral supplementation. Interestingly, 22 genes were shared between both contrasts—VTM_MG vs. VTM_LG and NoVTM_MG vs. NoVTM_LG. Among the genes, those related to lipid metabolism, including *ACSS2*, *FADS1*, *FADS2*, *FASN*, and *LPL*, were downregulated in the MG group regardless of the VTM treatment. Supporting our findings, decreased abundance of hepatic metabolites was reported by Crouse et al. in the livers of fetuses from MG compared to LG and from VTM compared with NoVTM groups [33]. Similarly, in a lipidomic study using the same fetuses as those evaluated here, Menezes et al. demonstrated that moderate rates of gain resulted in greater concentrations of hepatic polyunsaturated fatty acids and diacylglycerols and lower concentrations of monoacylglycerols [60]. Furthermore, they reported that 152 metabolites were significantly affected by the main effect of rate of gain, and, interestingly, greater abundances were observed in the livers of fetuses from cows under LG compared to MG [60]. In sheep, nutrient-restricted fetuses during late gestation were found to have decreased liver size and lipid metabolism [16].

The liver is a key organ controlling body energy metabolism [12]. Here we identified the PI3K-Akt signaling pathway as being over-represented in our gene-clustering approach and DEGs from the VTM_MG vs. VTM_LG contrast. PI3K-Akt is a nutrient-sensing pathway critical to biological processes, such as nutrient uptake, energy metabolism, and cell growth and differentiation [61,62]. We previously reported that this pathway was affected in 50-day-old fetuses from nutrient-restricted heifers [23]. Furthermore, placental cotyledons of nutrient-restricted ewes during the periconceptual period had increased activity of the PI3K/Akt pathway [63]. Many mineral-dependent enzymes are involved with energy and lipid metabolism [24,64,65]. Selenium is among the minerals modulating PI3K/Akt and energy metabolism [66]. Over-represented pathways affected by VTM supplementation included biosynthesis of unsaturated fatty acids, the PPAR signaling pathway, and fatty acid metabolism. The PPAR protein is sensitive to nutrient availability and plays a key role in tissue development [8]. Likewise, *FASN* encodes rate-limiting enzymes for long-chain fatty acid synthesis [67].

The upregulation of energy-metabolism-related genes in fetuses from heifers in the LG group combined with increased liver size may suggest compensatory adaptive mechanisms to uptake more nutrients [20]. The glucose-transport gene *SLC2A2* (*GLUT2*) was upregulated in the VTM_MG vs. VTM_LG comparison, whereas *SLC2A4* (*GLUT4*) was downregulated in NoVTM_MG vs. NoVTM_LG. Changes in response to reduced nutrient availability and compensatory growth are related to enhanced insulin secretion [68], likely explaining the over-representation of pathways such as insulin resistance and the insulin signaling pathway by the DEGs. Although these findings warrant further investigation of the mechanisms and long-term consequences for offspring performance, it has been shown that suboptimal maternal nutrition in ewes during early development of the fetal liver led to greater lipid accumulation within the liver following obesity during adulthood [17].

### 4.2. Periconceptual Maternal Mineral and Vitamin Supplementation Affect the Expression of Hepatic Fetal Genes Involved with Mineral Homeostasis and Amino Acid Transport

In addition to the effects of rate of weight gain, it is important to highlight the role of maternal vitamin and mineral supplementation on early fetal development. Although vitamins and minerals are required in small amounts, they are essential for proper fetal development, as they serve in structural, physiological, catalytic, and regulatory functions [54,65,69]. Furthermore, a growing number of studies has shown their role in modulating gene expression [65,70,71,72]. We previously reported that fetal liver mass was increased in VTM compared to NoVTM dams [29]. Furthermore, maternal vitamin and mineral supplementation (VTM) led to increased concentrations of Se, Cu, Mn, and Co in the livers of beef heifers and their fetuses [29]. Likewise, there was an increased abundance of Co, Zn, and Mo in allantoic and amniotic fluids at day 83 [30]. We also reported increased concentrations of histidine, aspartate, and 12 out of 14 neutral amino acids in the allantoic fluid of VTM-supplemented dams (*p* ≤ 0.05) [73]. Finally, fetal hepatic metabolites underlying amino acid metabolism were affected by maternal VTM supplementation [33].

The differentially expressed genes affected by the main effect of VTM supplementation included the metallothionein-coding genes *MT1A*, *MT1E*, and *MT2A*. Metallothioneins (MTs) are small, cysteine-rich, heavy-metal-binding proteins involved with protective stress responses [74]. The MT genes were upregulated in all contrasts, except VTM_MG vs. VTM_LG. The expression of MT genes is influenced by changes in trace-element availability [75]. Among the metal-transporter-coding genes, *SLC30A10* plays a role in body Mn levels [76] and was downregulated in the VTM_LG vs. NoVTM_LG comparison. Likewise, the selenoprotein-coding genes *SELENOI* and *SEPSECS* were upregulated in the VTM_MG vs. VTM_LG comparison. The mineral absorption pathway was over-represented by the DEGs from the VTM-supplemented groups. The MT coding genes underlie BP-related terms, such as the response to copper ions and detoxification of copper ions. Although there were no significant treatment effects on hepatic Zn levels, we found BP-related terms that were over-represented, such as Zn ion homeostasis and cellular response to Zn ions. Collectively, these findings show increased storage of specific trace minerals in the fetal liver, with consequences for gene expression and mineral transport. However, the interplay between specific trace minerals, their mechanisms of transport to the fetus, and their role in modulating gene expression and fetal programming warrant further studies.

We found over-represented BPs involved with amino acid (AA) transport of L-arginine, L-lysine, and ornithine. Essential AAs play key roles as regulators of metabolic and nutrient-sensing pathways, such as PPARs and AMPK signaling pathways [77]. According to Hussain et al., arginine is essential for growth and development, whereas ornithine plays a role in gene-expression regulation and protein synthesis [77]. Our research group has previously shown that vitamin and mineral supplements lead to increased levels of neutral AAs in the allantoic fluid of beef heifers [73]. Furthermore, we reported differential expression of nutrient-transport-coding genes in the placenta [31]. Lastly, Crouse et al. reported that fetal liver metabolites involved with the glycine, serine, and threonine pathway and the leucine, isoleucine, and valine pathway had greater abundances in the NoVTM_LG group [33]. Our results show that vitamin and mineral supplementation and rate of weight gain led to fetal metabolic adaptations and likely increased transport and use of AAs. However, questions related to maternal–fetal crosstalk and nutrient balance still remain.

## 5. Conclusions

This is the first study to investigate fetal hepatic gene expression in response to maternal vitamin and mineral supplementation and/or rate of weight gain during the periconceptual period. We have provided evidence that the maternal rate of gain during the first 83 days of pregnancy differentially regulated genes involved with metabolic-related pathways, including the PPAR, PI3K-Akt, AMPK, and insulin signaling pathways. These changes were accompanied by increased expression of genes involved with lipid and energy metabolism in fetuses from cows under a low rate of body-weight gain. Furthermore, mineral and vitamin supplementation led to the increased expression of genes involved with mineral homeostasis and the transport of amino acids. Collectively, our findings provide evidence of a potential compensatory attempt by the fetus to enhance energy metabolism under a low rate of weight gain. However, further research is needed to determine whether, and the extent to which, the changes in pathways affected during early gestation persist in the perinatal period and are translated into altered metabolic phenotypes in offspring.

## Figures and Tables

**Table 1:** *k*-means clusters and key over-represented pathways involved with fetal liver function.

Cluster	Number of Genes	Number of Pathways Over-Represented	Key Pathways *
A	702	15	Metabolic pathways Type I diabetes mellitus Retinol metabolism Cell adhesion molecules Arachidonic acid metabolism PPAR signaling pathway
B	577	6	ECM-receptor interaction Focal adhesion ABC transporters PI3K-Akt signaling pathway
C	357	–	–
D	364	15	Oxidative phosphorylation IL-17 signaling pathway Metabolic Ribosome Pathways of neurodegeneration

* FDR ≤ 0.05.

## Data Availability

All relevant data are included in the paper and in the Supplementary Materials. All sequencing data will be made publicly available in NCBI’s Gene Expression Omnibus upon acceptance of the paper.

## Supplementary Materials

The following supporting information can be downloaded at: https://www.mdpi.com/article/10.3390/ani13040600/s1, Table S1: Nutrient compositions of total mixed rations and supplements provided to beef heifers. Table S2: Summary of RNA sequencing, mapping statistics, and treatments. Table S3: Differentially expressed genes, KEGG pathways, and biological process between NoVTM_MG and NoVTM_LG. Table S4: Differentially expressed genes, KEGG pathways, and biological process between VTM_LG and NoVTM_LG. Table S5: Differentially expressed genes, KEGG pathways, and biological process between VTM_LG and NoVTM_MG. Table S6: Differentially expressed genes, KEGG pathways, and biological process between VTM_MG and NoVTM_LG. Table S7: Differentially expressed genes, KEGG pathways, and biological process between VTM_MG and NoVTM_MG. Table S8: Differentially expressed genes, KEGG pathways, and biological process between VTM_MG and VTM_LG. Table S9: Overlapping differentially expressed genes among contrasts. Table S10: Overlapping KEGG pathways among contrasts. Table S11: *k*-means clustering analysis of fetal liver genes at day 83 of pregnancy. Table S12: Over-represented KEGG pathways from *k*-means clustering analysis of fetal liver genes at day 83 of pregnancy (FDR < 0.05).
